# Prostate cancer dural metastasis resembling a meningioma

**DOI:** 10.1002/ccr3.5601

**Published:** 2022-04-04

**Authors:** Hitomi Shimada, Masaki Tago

**Affiliations:** ^1^ Department of General Medicine Saga University Hospital Saga Japan; ^2^ Shimada Hospital of Medical Corporation Chouseikai Saga Japan

**Keywords:** bone thickening, brain tumor resembling meningioma, dural metastasis, prostate cancer

## Abstract

CT images of a 56‐year‐old man with headache showed a meningioma‐like mass in the occipital region. The tumor was well‐defined and non‐uniform with bone thickening and no internal calcification. Eventually, he was diagnosed on the basis of histopathology and immunostaining findings as having a dural metastasis from a prostate cancer.

## Graphical abstract

In men, brain tumors that resemble meningiomas on CT images and are associated with bone thickening and rapid growth should be strongly suspected of being dural metastases from prostate cancer and therefore be evaluated quickly.

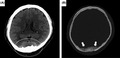

## 1 CASE

**FIGURE 1:**
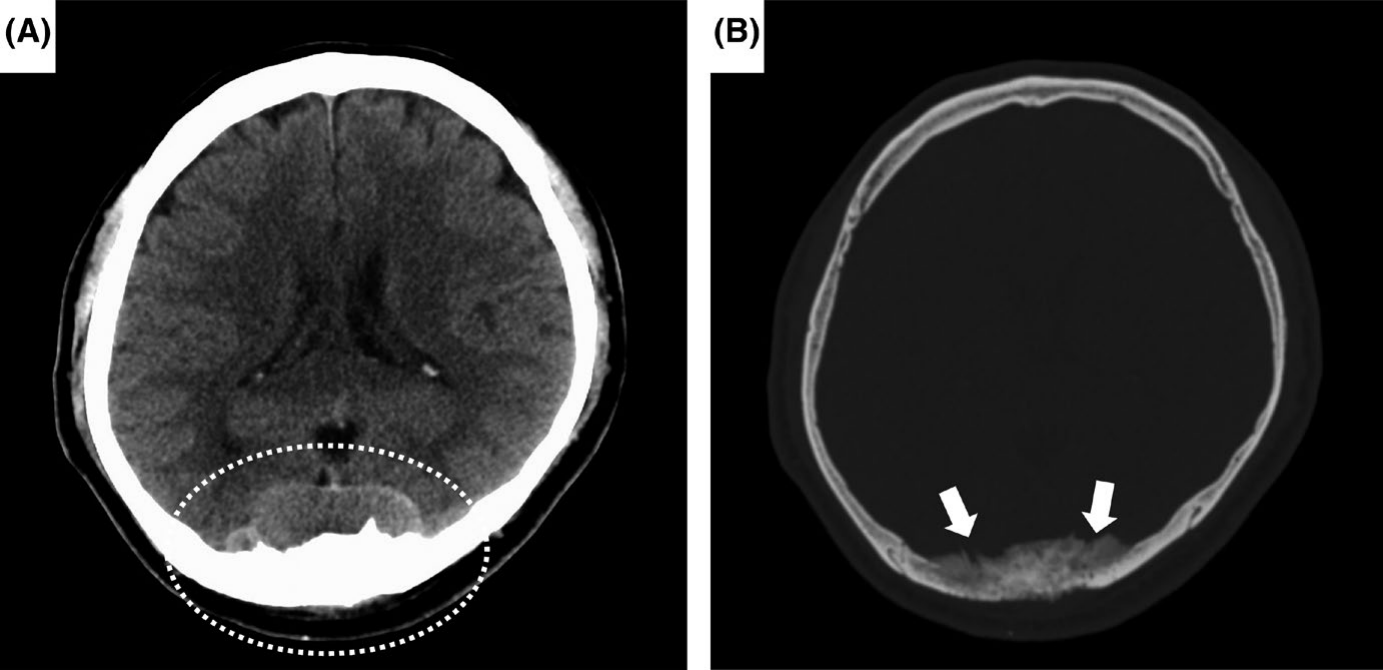
Head CT without contrast enhancement. (A) Axial brain image showing a 54 × 15 × 64 mm mass in the occipital region (A, dashed circle). The tumor is well‐defined and non‐uniform and has no internal calcification. The lesion is located in the epidural space and occipital bone and likely extends into the subdural and subarachnoid spaces. The brain is compressed; however, there are no abnormalities in the brain parenchyma. (B) Axial bone image showing thickening of the occipital bone (B, arrows). In addition, there is a bone lesion with irregular margins in which the CT value is lower than it is in the normal bone cortex

**FIGURE 2:**
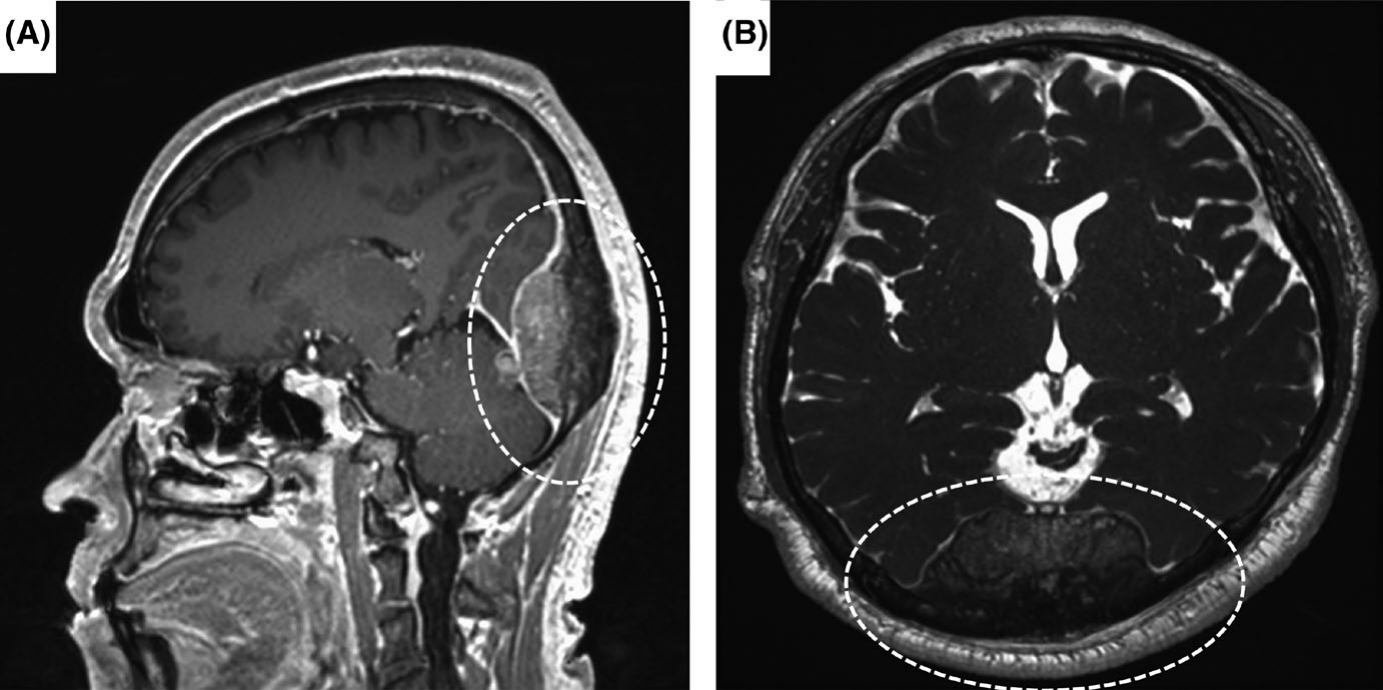
Cranial magnetic resonance imaging with contrast enhancement. (A) Sagittal T1‐weighted magnetization‐prepared rapid gradient echo image. (B) Axial T2‐weighted constructive interference in a steady‐state image. Magnetic resonance images showing a well‐defined mass in the mid‐occipital region from the occipital bone to the epidural space, with slightly low intensity on T1‐weighted images (A), slightly heterogeneous and low intensity at the margins, and mildly high intensity at the center on T2‐weighted images (B). The lesion in the bone is low intensity in both T1‐ and T2‐weighted images

A 56‐year‐old man reported having a headache and right temporal pain for 3 months. He was alert and had no fever, abnormal neurological findings, or any urinary or prostatic symptoms. Head CT revealed a 54 × 15 × 64 mm mass in the occipital region (Figure 1A) with adjacent bone thickening (Figure 1B). Cranial MRI with contrast enhancement showed a tumor in the epidural space extending into the skull and medial to the dura (Figure 2). These findings are strongly suggestive of meningioma, and the tumor was resected without further systemic examination. However, histopathological examination revealed a growth pattern that differed from that characteristic of a meningioma. Immunostaining showed prostate‐specific antigen (PSA)‐positive and alpha‐methylacyl‐CoA racemase‐positive tumor cells. Furthermore, the serum PSA was significantly elevated at 832.4 ng/mL. Thus, a dural metastasis from prostate cancer was diagnosed. Postoperative CT with contrast enhancement and bone scintigraphy revealed multiple metastases in the pelvic lymph nodes and bones. The patient was started on postoperative combined androgen blockade therapy to which he responded well, his PSA improving to 0.054 ng/mL by 4 months after the operation.

Intracranial metastases from prostate cancer are rare. However, dural metastases, which occur less frequently with other cancers, are relatively common in prostate cancer. In addition, dural metastases from prostate cancer often resemble meningiomas on imaging studies.^1^ In men, brain tumors that resemble meningiomas and have associated bone thickening and rapid growth, together with absence of neurological abnormalities or calcification on CT, should be strongly suspected of being dural metastases from prostate cancer and should therefore be evaluated quickly.^2^

## CONFLICT OF INTEREST

The authors state that they have no conflict of interest.

## AUTHOR CONTRIBUTIONS

HS was involved in the literature search, study conception, drafting of the manuscript, and clinical care of the patient. MT was involved in the literature search, study conception, and drafting and revision of the manuscript.

## ETHICAL APPROVAL

This report conforms to the provisions of the Declaration of Helsinki in 1995 (as revised in Brazil 2013).

## CONSENT

Written informed consent to publish this report was obtained from the patient in accordance with the journal's patient consent policy.

## Data Availability

The data that support the findings of this study are available from the corresponding author upon reasonable request.
